# Supplementary material for: Cysteine-enabled cleavability to advance cross-linking mass spectrometry for global analysis of endogenous protein-protein interactions
Source: Nat Commun. 2025 Dec 12;16:11093. doi: 10.1038/s41467-025-66023-0 (PMC12701074; doi:10.1038/s41467-025-66023-0)
Supplement: Supplementary file 1 — Supplementary Information [file 41467_2025_66023_MOESM1_ESM.pdf]

SUPPLEMENTAL FIGURES

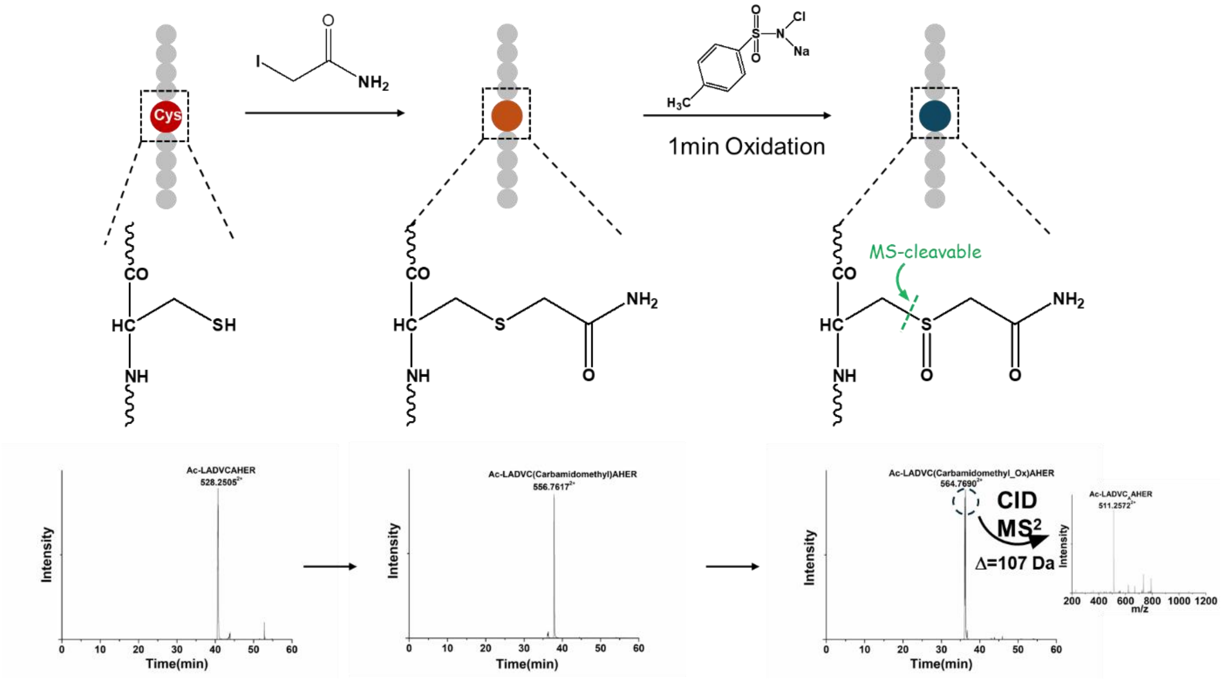

Supplementary Fig. 1. Oxidation-induced MS-cleavability of carbamidomethylated cysteine. MS^2 fragmentation of synthetic peptide Ac-LR9, carbamidomethylated Ac-LR9, and oxidized carbamidomethylated Ac-LR9, respectively.

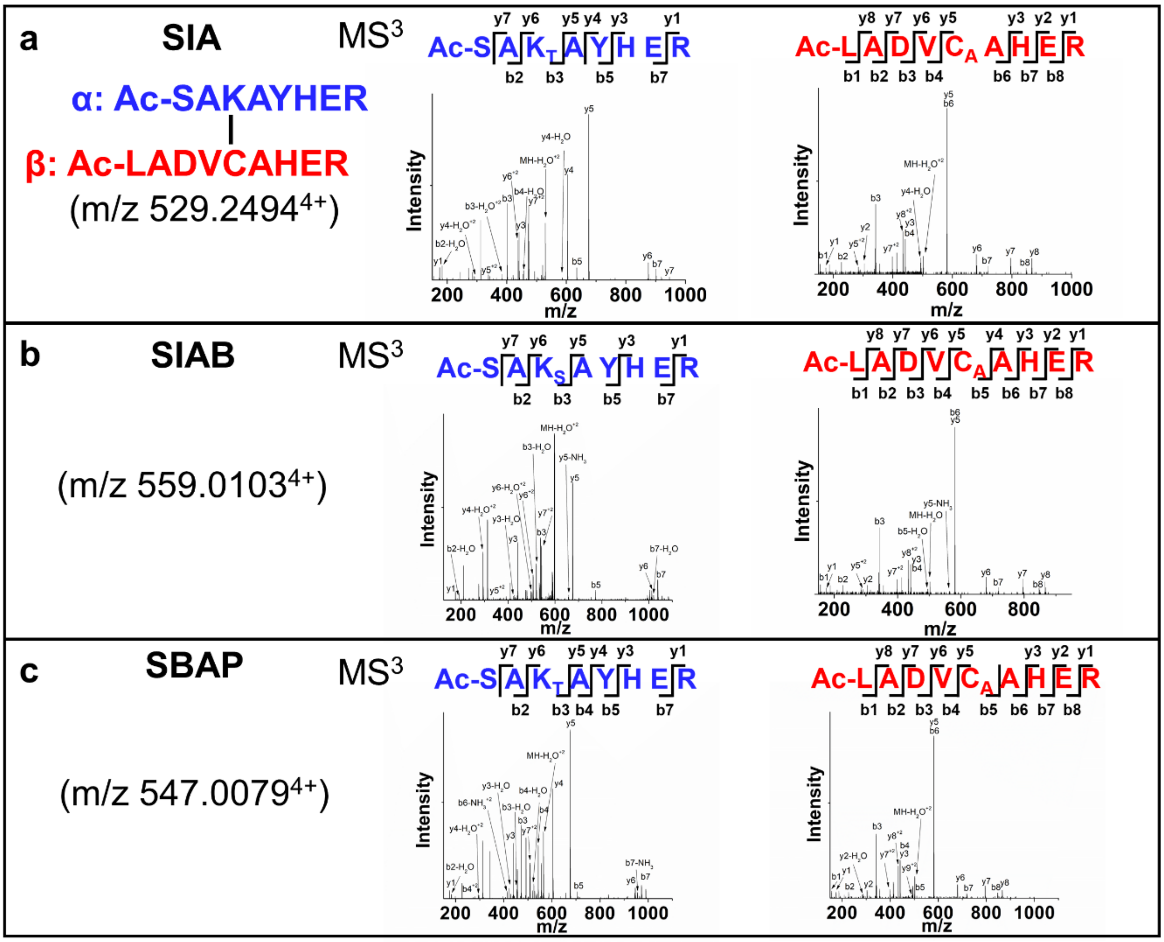

Supplementary Fig. 2. MS^3 analyses of the Ac-SR8-Ac-LR9 cross-linked peptides. MS^3 spectra of the $\alpha_{S/T}$ and β_A fragment ions that were detected in their corresponding MS^2 spectra shown in Fig. 2a using: **a** SIA, **b** SIAB and **c** SBAP cross-linkers.

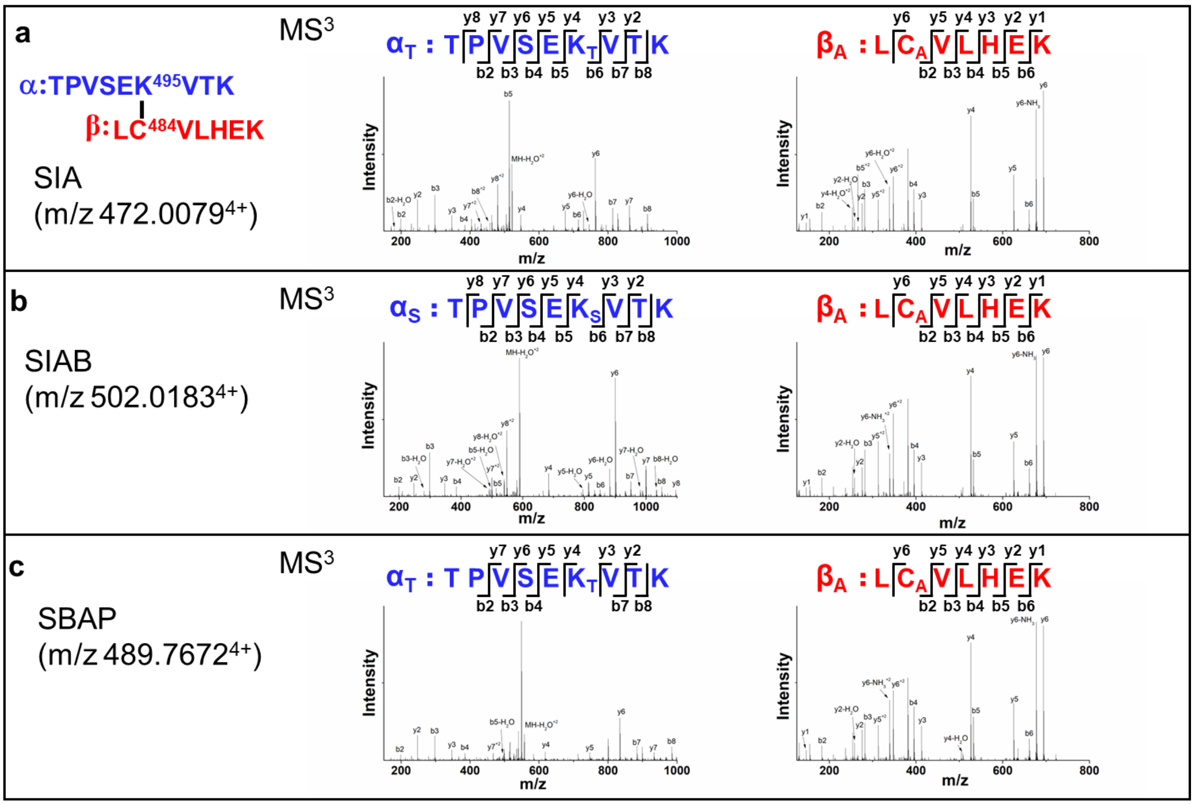

Supplementary Fig. 3. MS^3 analyses of representative K-C cross-linked peptides of BSA signifying a linkage between K495 and C484. MS^3 spectra of the $\alpha_{S/T}$ and β_A fragment ions that were detected in their corresponding MS^2 spectra shown in Fig. 2b using: **a** SIA, **b** SIAB and **c** SBAP cross-linkers.

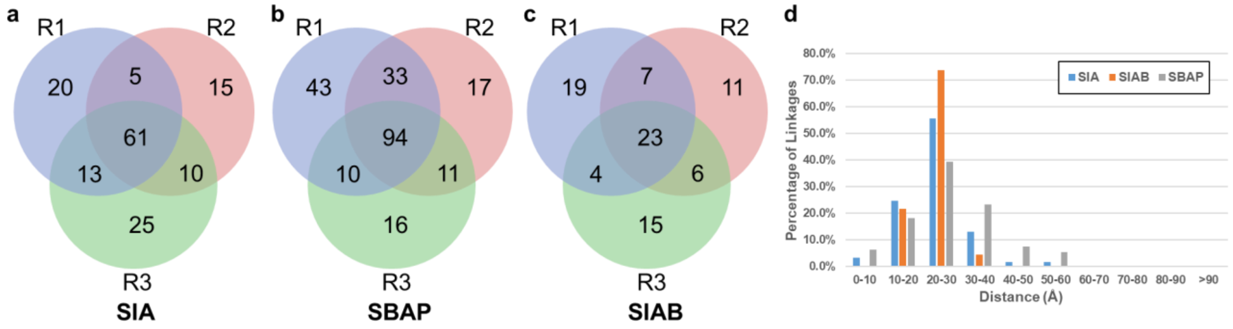

Supplementary Fig. 4. Overlap of BSA K-C linkages identified across three replicates for each linker: **a** SIA, **b** SBAP, and **c** SIAB. **d** Distance distribution of SIA, SIAB, SBAP cross-linked linkages

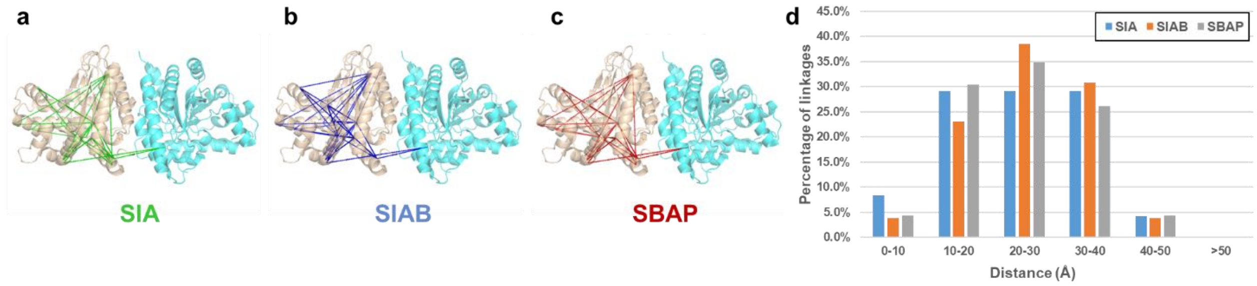

Supplementary Fig. 5. 3-D XL-maps of ALDOA revealed by a SIA, **b** SIAB, and **c** SBAP cross-linking. **d** Distance distribution of SIA, SIAB, SBAP cross-linked linkages

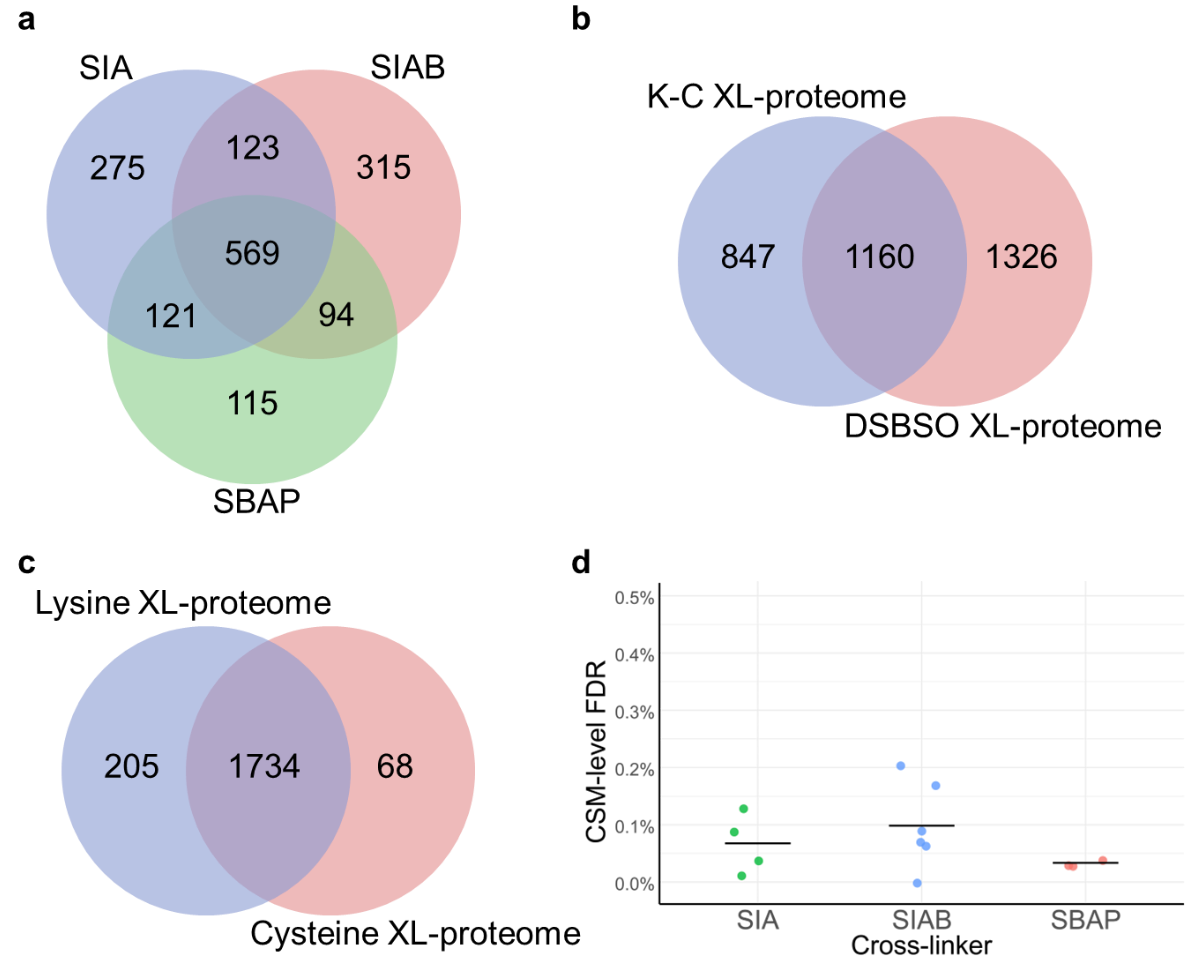

Supplementary Fig. 6. Comparison of K-C XL-proteomes and associated CSM-level FDR evaluation. **a** Overlap of SIA, SBAP and SIAB XL-proteomes identified from MS^3 analysis. **b** Overlap of the combined K-C XL-proteome and K-K (DSBSO) XL-proteome. **c** Overlap of XL-proteins identified with cross-linked lysine and cysteine residues. **d** CSM-level FDRs for MS^n-based identification of SIA, SIAB, and SBAP cross-linked peptides across biological replicates. SIA: 4 replicates; SIAB: 6 replicates; and SBAP: 3 replicates.

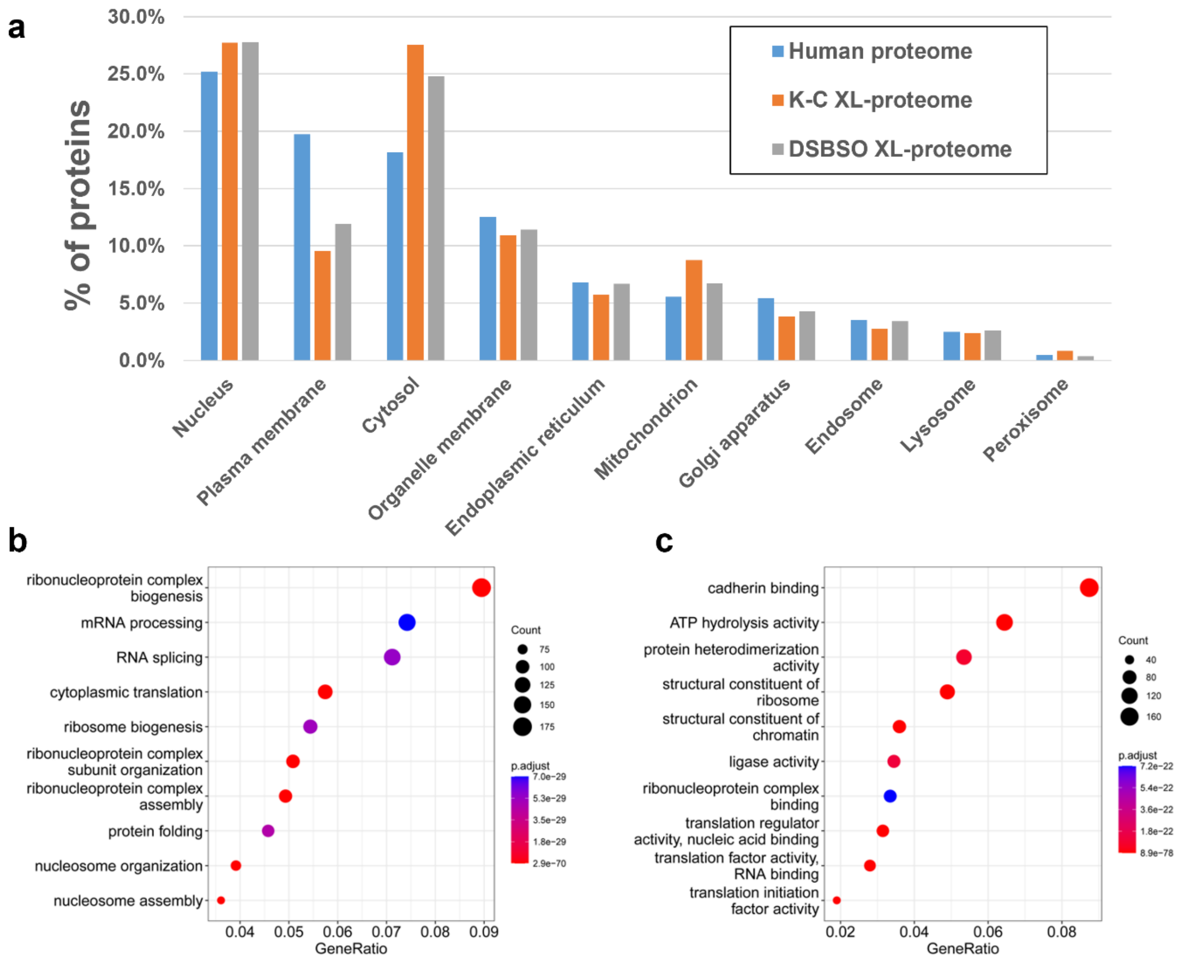

Supplementary Fig. 7. GO enrichment analysis. **a** Cellular compartment enrichment analysis of K-C XL-, DSBSO XL- and Human-proteomes. GO enrichment analysis of K-C XL-proteome for **b** Biological processes and **c** Molecular functions.

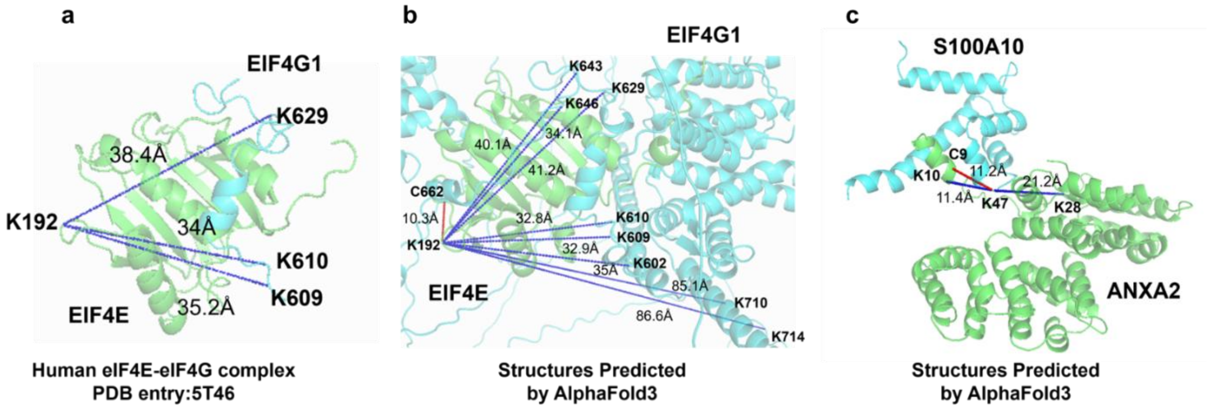

Supplemental Fig. 8. Structural mapping of the identified K-C cross-links and potential K-K cross-links from the two selected interactions. a Structural mapping of the EIF4E-EIF4G complex with partial sequences (PDB ID: 5T46, EIF4E:31-206, EIF4G1:608-642) to illustrate the distances between EIF4E:K192 and EIF4G1:K609, K610, and K629. **b** Analysis of the AF-predicted structure of the EIF4E-EIF4G1:C662 complex to map the identified EIF4E:K192-EIF4G1:C662 cross-link (shown in red) and determine the distances between EIF4E:K192 and the eight lysines of nearby C662 of EIF4G1 (shown in blue). **c** Analysis of the AF-predicted structure of the S100A10-ANXA2 complex to map the identified K-C cross-link (i.e. S100A10:K47-ANXA2:C9) (shown in red) and illustrate the proximity between S100A10:K47 and the lysine residues (i.e. K10, K28) nearby C9 of ANXA2 (shown in blue).

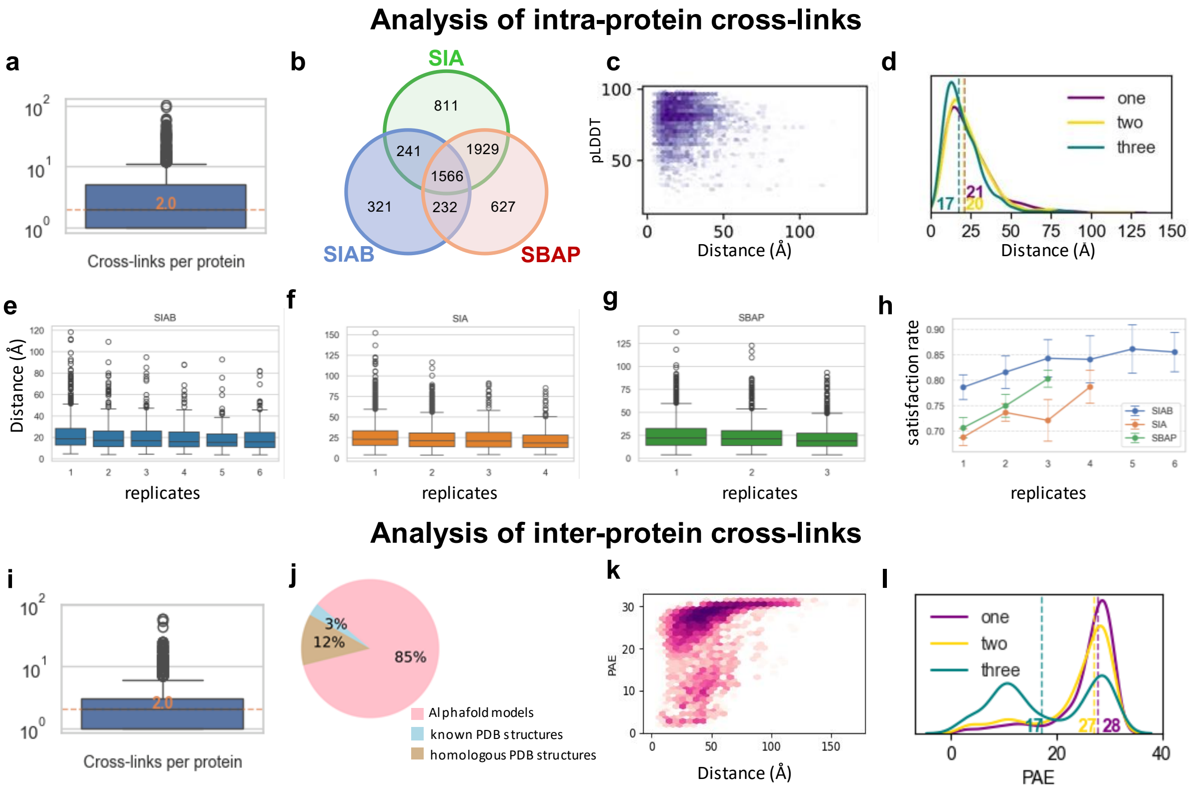

Supplementary Fig. 9. Structural analysis of K-C cross-links. a Number of intra cross-links per protein. **b** Venn diagram comparing cross-links identified across three K-C cross-linkers. **c** Mapped cross-link distances vs. pLDDT. **d** Distribution of K-C cross-link mapped distances against PDB structures depending on identification frequency across different K-C cross-linkers. **e-g** Distribution of cross-link distances as a function of the number of biological replicates. **h** Satisfaction rate (% of cross-links with distance <30 Å), plotted with 95% confidence intervals as a function of biological replicates. **i** Number of inter cross-links per protein-protein interaction. **j** Structural coverage of the inter-protein cross-links. **k** Distribution of distances between cross-linked amino acids in the AlphaFold-multimer models vs. PAE. **l** Distribution of PAE values between cross-linked amino acids in the AlphaFold-multimer models according to identification frequency across different K-C cross-linkers.

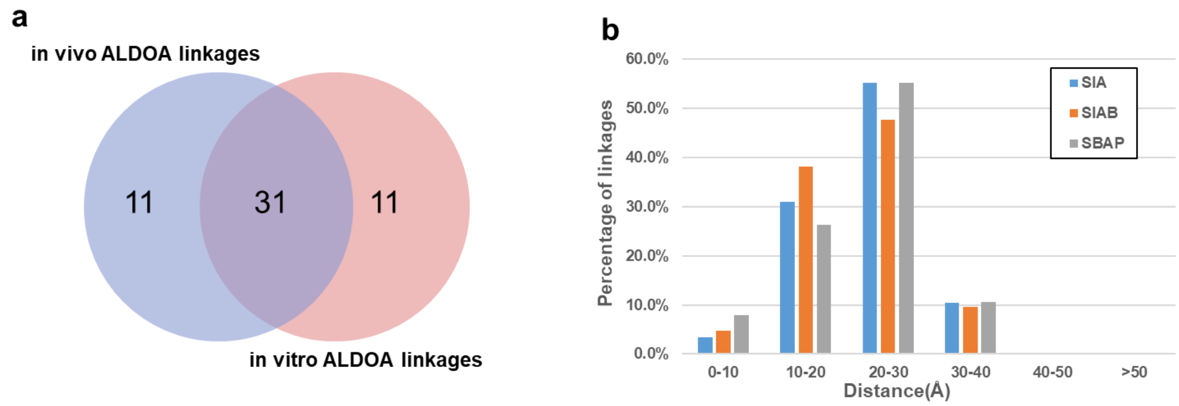

Supplementary Fig. 10. Comparison of ALDOA K-C linkages identified in in vivo and in vitro experiments. a Overlap of total ALDOA K–C cross-links identified from in vivo and in vitro cross-linking experiments. **b** Distribution of Cα–Cα distances for ALDOA K–C cross-links identified in vivo using SIA, SIAB, and SBAP cross-linkers.

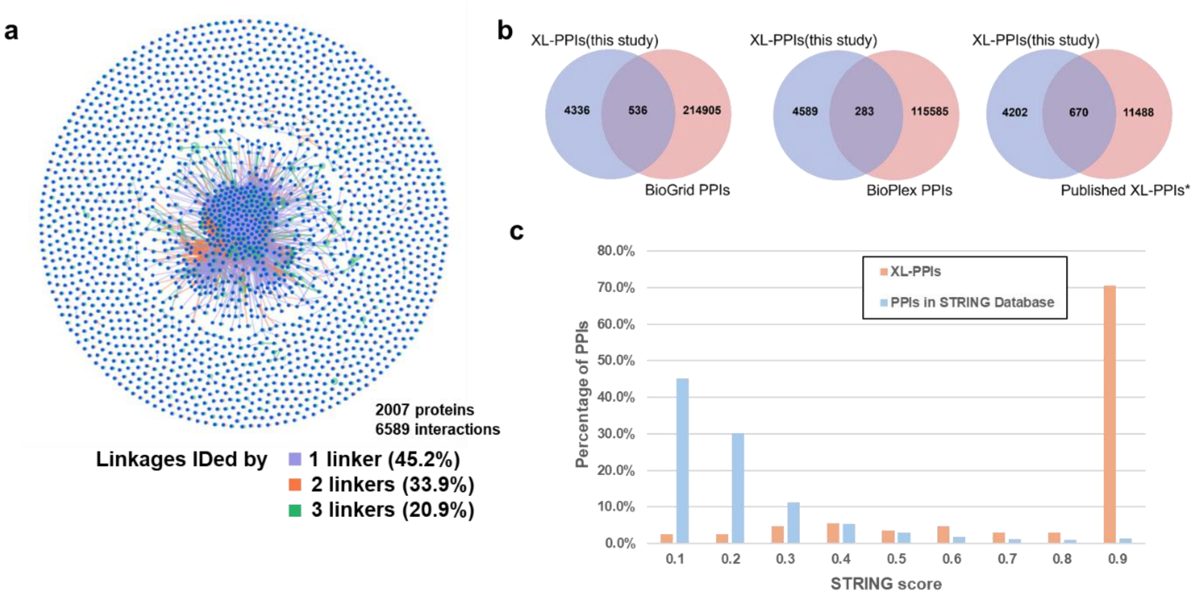

Supplementary Fig. 11. Analysis of K-C XL-PPIs. a K-C XL-PPI network of HEK 293 cells composed of 2,007 proteins and 6,589 interactions. **b** Comparison of the XL-PPIs from this study against BioGrid, BioPlex databases and published XL-PPI data[1,2,3,4,5,6,7] **c** Distribution of high confident identified PPIs with associated STRING Scores.

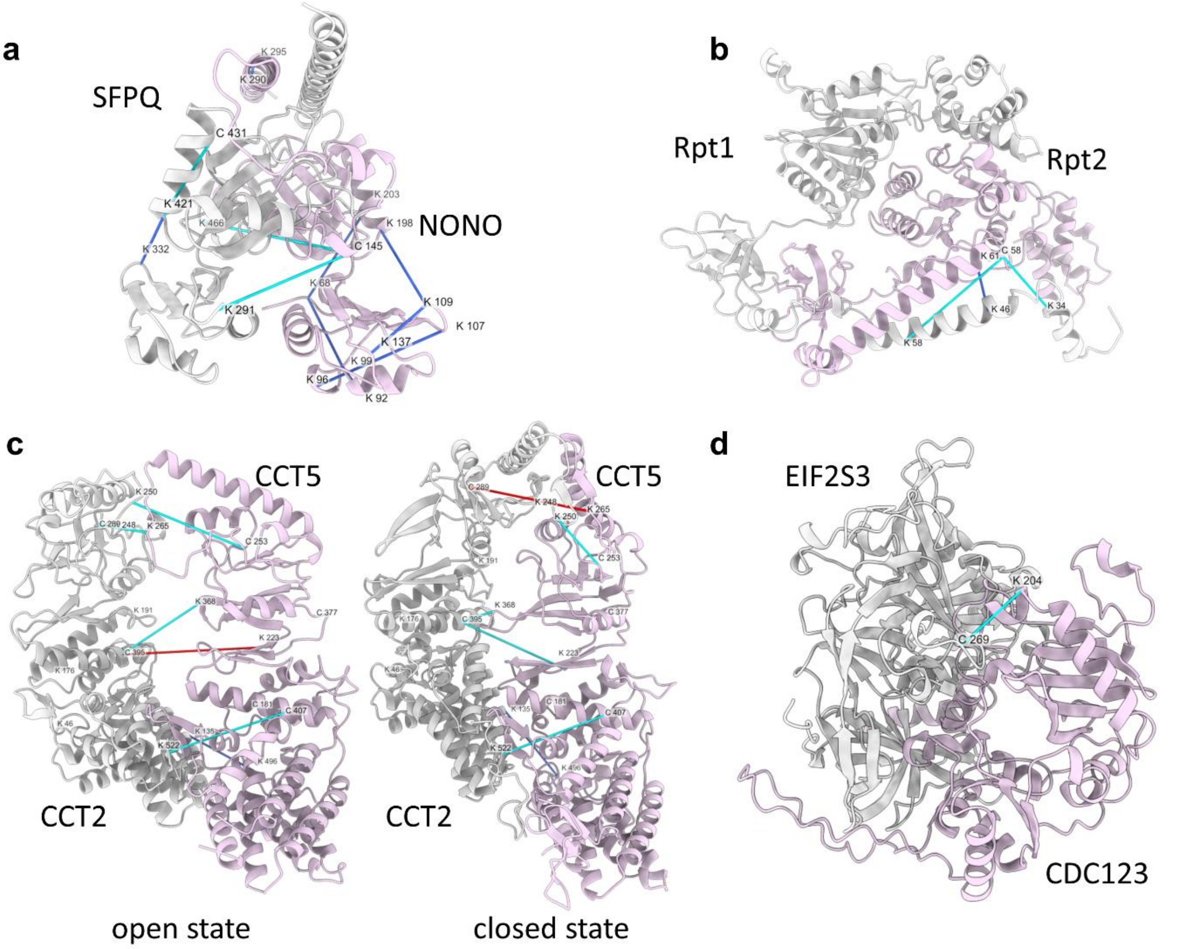

Supplementary Fig. 12. XL structural mapping. Mapping of satisfied K-C and K-K (DSBSO) linkages (<30 Å) in the structures of **a** SFPQ-NONO (PDB entry: 7PU5), **b** Rpt1-Rpt2 (PDB entry: 7W37), **c** CCT2-CCT5 (PDB entry: 7WU7 open state, 7NVL closed state), and **d** EIF2S3-CDC123 (AlphaFold predicted structure). (teal: K-C linkages, blue: K-K linkages, red: conformation-dependent K-C linkages)

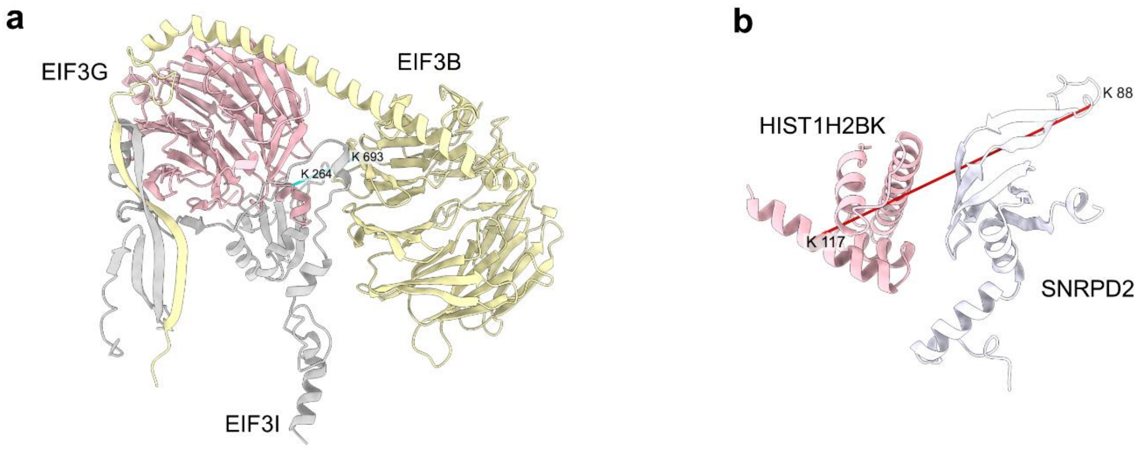

Supplementary Fig. 13. XL structural mapping. XL mapping of **a** EIF3G (pink), EIF3I (silver), and EIF3B (gold) with one DSBSO K-K cross-link, **b** HIST1H2BK (pink) and SNRPD2 (silver) with the one DSBSO K-K cross-link. Note: blue for satisfied and red for violated cross-links.

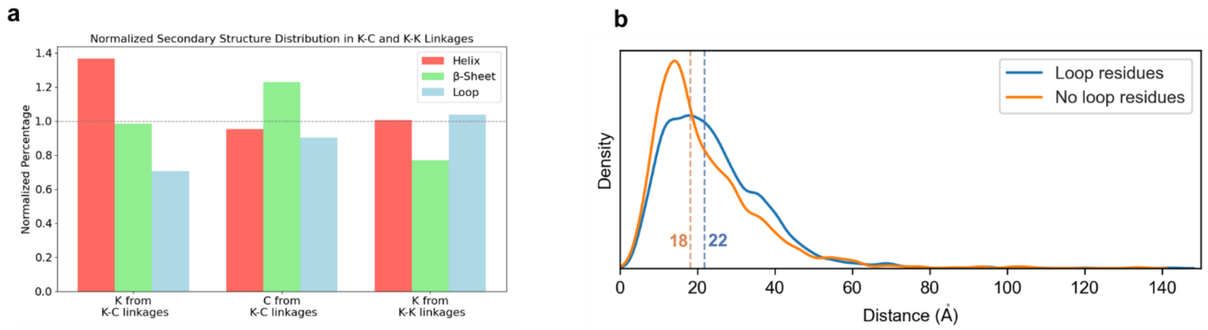

Supplementary Fig. 14. Secondary structure localization of cross-linked residues for **a** Lysine and Cysteine from K-C cross-links and Lysine from K-K (DSBSO) cross links. **b** Distance distribution of K-C linkages cross-linked at loop versus non-loop residues.

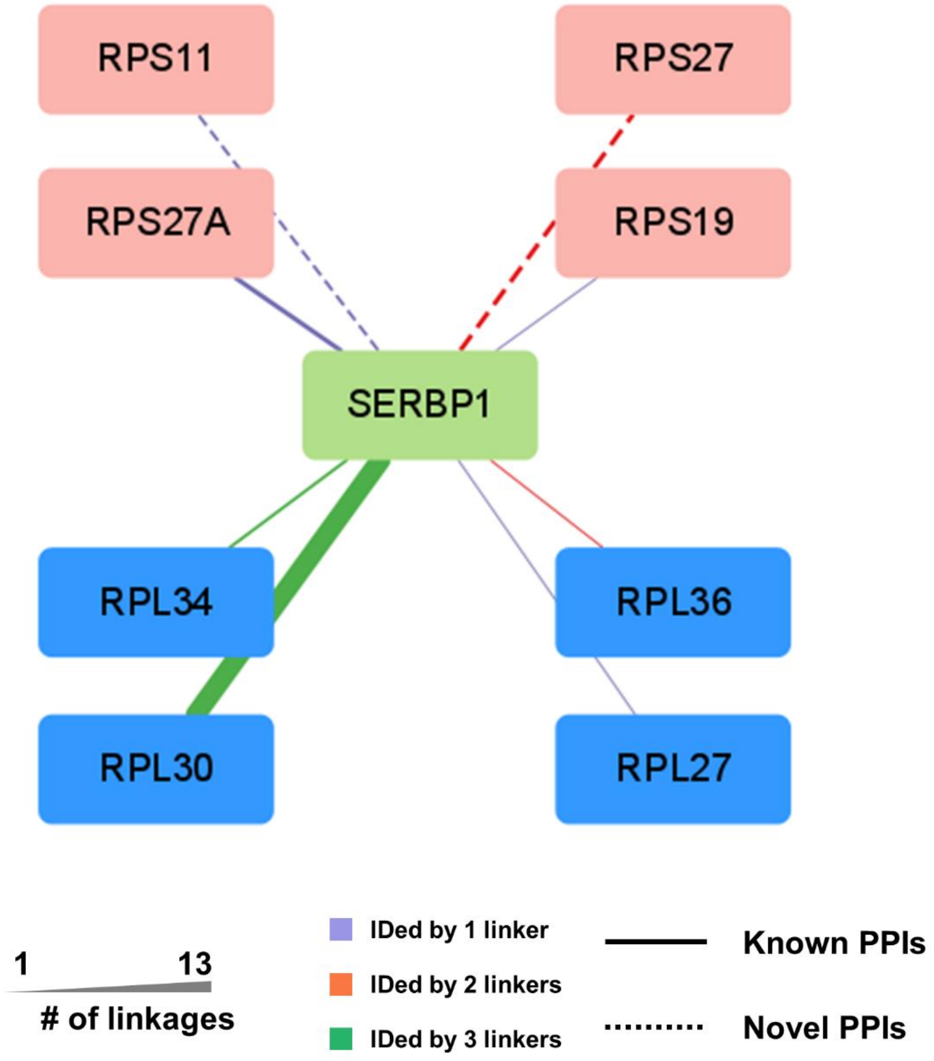

Supplementary Fig. 15. XL-PPI subnetwork of the SERBP1-ribosomal complex revealed by K-C XL-MS analyses. Previously reported PPIs shown with solid edges; novel PPIs shown with dashed edges.

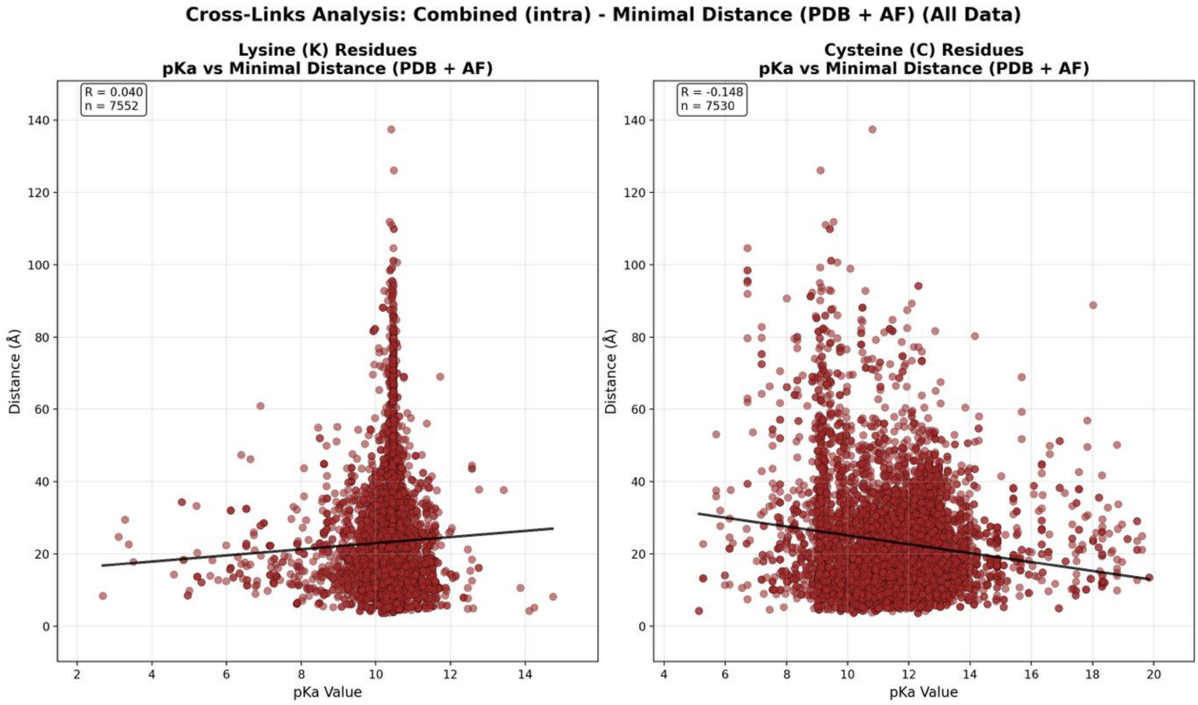

Supplementary Fig. 16. Distribution of pKa values for cross-linked lysines (left) and cysteines (right) as calculated by PROPKA 3.1[8]

SUPPLEMENTARY METHODS

In Vivo Cross-linking of HEK 293 Cells

The HEK 293 cells were cultured in Dulbecco's modified Eagle's medium (DMEM) supplemented with 10% fetal bovine serum (FBS) and 1% penicillin-streptomycin until 90% confluence as previously described[1]. After washing with PBS buffer twice, each plate of cells were resuspended in 1mL of PBS containing 2mM SIA, 1mM SIAB or 2mM SBAP. The cells were cross-linked for 30 min with rotation at 37ºC or RT(SIAB) in dark. The cells were spun down and washed with PBS buffer to remove excess linker. After capping with 50mM IAA for 30min, the SIA cross-linked cells were lysed by a two-step protein extraction method as previously reported with minor changes[1]. Briefly, cells were first lysed with denaturing buffer No.1 (8M urea, 50mM Tris HCl, 0.5% NP-40, 50mM IAA, pH 7.0). The supernatant was collected after centrifugation at 14,000g and the protein concentration was measured by Bradford. Then, 1mg of protein were subjected to a FASP digestion. The remaining cell pellet after centrifugation was resuspended in buffer No.2 (8M urea, 25mM ABC, 50mM IAA, pH 8) for directly in solution digestion. The desalted peptides were dissolved in 30% ACN/0.1% FA before loaded onto a SuperdexTM Peptide 3.2/300 SEC column for separation. The two SEC fractions containing cross-linked peptides were collected, dried and oxidized as described above. The oxidized peptides were dissolved in 160 μL of ammonia water (pH10) and subjected to HpHt fractionation separately. The SIAB or SBAP cross-linked cells were only lysised by buffer No.1. After lysing, the digestion and fraction methods were the same as that applied for SIA cross-linked proteins. We performed four replicates of SIA, six replicates of SIAB (three at room temperature and three at 37°C), and three replicates of SBAP analyzed by MS^3. Additionally, we included two more SIA replicates (R3 and R4), three room-temperature SIAB replicates, and three SBAP replicates analyzed by MS^2.

Protein Digestion

Cross-linked proteins were initially transferred onto a 30 kDa FASP centrifugal filter and subsequently washed with 8 M urea to remove contaminants. The proteins were then denatured and subjected to reduction using 2 mM TCEP for 30 minutes, followed by alkylation with 10 mM iodoacetamide for 30 minutes in the dark to prevent cysteine oxidation. For ALDOA, the protein was alkylated with 10 mM iodoacetamide for 30 minutes in the dark without TCEP reduction. After an additional wash with 25 mM ammonium bicarbonate, the proteins were

reconstituted in a solution containing 60 µL of 8 M urea and 25 mM ammonium bicarbonate, along with 25 µL of 25 mM ammonium bicarbonate. Lys-C was then added at an enzyme-to-protein ratio of 1:100, and the mixture was incubated at 37°C for 4 hours to initiate proteolysis. Subsequently, the urea concentration was diluted to 1.5 M to facilitate trypsin digestion at an enzyme-to-protein ratio of 1:50, which was carried out at 37°C overnight. The digested peptides were acidified by adding trifluoroacetic acid (TFA) to a final concentration of 0.1%, followed by desalting using a Sep-Pak C18 cartridge. Finally, the eluents were vacuum-dried and stored at -80°C before size-exclusion chromatography (SEC) fractionation.

SEC-HpHt Fractionation of Cross-linked Peptides

Cross-linked peptides were separated by size exclusion chromatography coupled with high pH reverse phase tip fractionation (SEC-HpHt) following a previously described method[2]. Briefly, 250 µg of desalted peptides were dissolved in 30% ACN/0.1% TFA and loaded onto a Superdex™ Peptide 3.2/300 SEC column for separation. Two SEC fractions containing cross-linked peptides were collected, dried, and oxidized for HpHt fractionation. For HpHt separation, a 200 µL pipette tip was blocked using C8 membrane (Empore, 3M) and filled with 5 mg C18 solid phase (3 µm, Durashell, Phenomenex). The tip was sequentially equilibrated with 90 µL of methanol, ACN, and ammonia water (pH 10). The dissolved peptides were then loaded onto the tip and centrifuged at 1,200 rpm until the liquid level neared the beads. After washing with an additional 90 µL of ammonia water (pH 10), peptides were eluted with increasing concentrations of ACN in ammonia water (6%, 9%, 12%, 15%, 18%, 21%, 25%, 30%, 35%, and 50%). The 25%, 30%, 35%, and 50% fractions were combined into the 6%, 9%, 12%, and 21% fractions, respectively. The resulting six fractions were vacuum dried and stored at -80°C until MS analysis.

LC-MS^n analysis

LC MS^n analysis of SEC-HpHt fractions were carried out using an UltiMate 3000 RSLC coupled with an Orbitrap Fusion Lumos mass spectrometer. Samples were loaded onto a 50 cm x 75 µm Acclaim PepMap C18 column and separated over a 120 min gradient of 4% to 25% acetonitrile at a flow rate of 300 nL/min. The top 4 data-dependent MS^3 acquisition method was used for the identification of K-C cross-linked peptides. Ions with charge of 4^+ to 8^+ in the MS^1 scan were selected for MS^2 analysis. The top 4 most abundant fragment ions in MS^2 scan were further fragmented by CID with a collision energy of 35%.

Identification of Cross-linked Peptides by MS^n analysis

MS^3 spectra were extracted using PAVA (UCSF) and analyzed using Batch-Tag within Protein Prospector (v.6.3.5) by searching against the SwissProt human database (in vivo experiments, version 2021.10.02; 20,387 entries), Bos Taurus database (BSA, version 2019.04.08; 6,006 entries) or Oryctolagus Cuniculus database (ALDOA, version2024.08.05; 978 entries) with a random concatenated decoy database. The mass tolerances were set to ±20 ppm for precursor ions and 0.6 Da for fragment ions. Trypsin was designated as the proteolytic enzyme, allowing for up to three missed cleavages. A maximum of four variable modifications were permitted, including carbamidomethylation and oxidized carbamidomethylation of cysteine, methionine oxidation, N-terminal acetylation, and the conversion of N-terminal glutamine to pyroglutamic acid. For SIA-modified peptides, three additional modifications were included: unsaturated thiol (+C_2SO, +71.967 Da) and sulfenic acid (+$C_2H_2O_2S$, +89.9776 Da) on uncleaved lysines and protein N-termini, as well as alkene modification (-SH_2, -33.9877 Da) on cysteine residues. For SBAP, additional modifications comprised unsaturated thiol (+$C_5H_5O_2NS$, +143.0041 Da) and sulfenic acid (+$C_5H_7O_3NS$, +161.0147 Da) on uncleaved lysines and protein N-termini, along with alkene modification (-SH_2, -33.9877 Da) on cysteine. For SIAB, the modifications included sulfenic acid (+$C_9H_7O_3NS$, +209.0147 Da) on uncleaved lysines and protein N-termini, and alkene modification (-SH_2, -33.9877 Da) on cysteine. Cross-linked peptides were identified, summarized, and validated using the in-house software XL-Tools, based on the Protein Prospector search results and MS^n data. For a cross-link to be successfully identified, two linear peptide constituents must be identified from the MS^3 within the same MS^2 scan, carry unique cross-linker modifications due to characteristic fragmentation, and satisfy a defined mass relationship to their parent ion measured in MS^1 scan[9]. FDRs at the CSM level were calculated based on a target-decoy approach[10].

LC-MS^2 analysis

LC MS^2 analysis of SEC-HpHt fractions was performed using the same LC parameters as described above for LC MS^3 analyses. For MS^2 analysis, each scan cycle consists of an MS^1 scan acquired in the Orbitrap, followed by data-dependent Orbitrap MS^2 scans on MS^1 ions with charge $4\text{-}8^+$ for 3 seconds at top speed. MS^1 scans were acquired at 60,000 resolution across a scan range from 375-1500 m/z with AGC target 5e5 and a maximum injection time of 50ms. Ions selected from MS^1 were fragmented using stepped-energy HCD (NCE 27±6%) and

acquired in MS^2 at 30,000 resolution with AGC target 5e4 and maximum injection time 100ms. Dynamic ion exclusion was set to 30 seconds with mass tolerance of ±8 ppm.

Identification of Cross-linked Peptides by stepped-HCD MS^2 analysis

MS^2 spectra were extracted using PAVA and analyzed using Batch-Tag within a developmental version of Protein Prospector (v.6.4.29) by searching against a randomly concatenated decoy-containing database consisting of 3,359 proteins that were identified from DSSO(K-K)[2], DBrASO(C-C)[3] in vitro cross-linking studies and SIA MS^3 searches. The mass tolerances were set to ±15 ppm for precursor ions and ±20 ppm for fragment ions. Trypsin was designated as the proteolytic enzyme, allowing for up to two missed cleavages. A maximum of two variable modifications were permitted, including carbamidomethylation and oxidized carbamidomethylation of cysteine, methionine oxidation, N-terminal acetylation, and the conversion of N-terminal glutamine to pyroglutamic acid. The FDR was set to 1% at total CSMs level. For SIA cross-link analysis, an MS-cleavable cross-link was specified between lysine and cysteine residues with elemental composition C_2O_2 (55.9898 Da) and fragmenting into an unsaturated thiol (+C_2SO, +71.967 Da) on lysine and an alkene modification (-SH_2, -33.9877 Da) on cysteine residues. For SBAP, the cross-link bridge mass was specified as $C_5H_5O_3N_1$ (127.0269 Da), with fragmentation resulting in an unsaturated thiol (+$C_5H_5O_2NS$, +143.0041 Da) on lysine and an alkene modification (-SH_2, -33.9877 Da) on cysteine residues. For SIAB, the cross-link bridge mass was specified as $C_9H_7N_1O_3$ (175.0269 Da) with MS fragmentation resulting in a sulfenic acid (+$C_9H_7O_3NS$, +209.0147 Da) on lysine and an alkene modification (-SH_2, -33.9877 Da) on cysteine residues.

SUPPLEMENTARY REFERENCES

1. Wheat A, et al. Protein interaction landscapes revealed by advanced in vivo cross-linking–mass spectrometry. *Proceedings of the National Academy of Sciences* **118**, e2023360118 (2021).

2. Jiao F, Yu C, Wheat A, Wang X, Rychnovsky SD, Huang L. Two-dimensional fractionation method for proteome-wide cross-linking mass spectrometry analysis. *Analytical chemistry* **94**, 4236-4242 (2022).

3. Jiao F, Salituro LJ, Yu C, Gutierrez CB, Rychnovsky SD, Huang L. Exploring an alternative cysteine-reactive chemistry to enable proteome-wide PPI analysis by cross-linking mass spectrometry. *Analytical chemistry* **95**, 2532-2539 (2023).

4. Liu F, Lössl P, Scheltema R, Viner R, Heck AJ. Optimized fragmentation schemes and data analysis strategies for proteome-wide cross-link identification. *Nature communications* **8**, 15473 (2017).

5. Yugandhar K, et al. MaXLinker: proteome-wide cross-link identifications with high specificity and sensitivity. *Molecular & Cellular Proteomics* **19**, 554-568 (2020).

6. Bartolec TK, et al. Cross-linking mass spectrometry discovers, evaluates, and corroborates structures and protein–protein interactions in the human cell. *Proceedings of the National Academy of Sciences* **120**, e2219418120 (2023).

7. Jiao F, et al. DSBSO-Based XL-MS Analysis of Breast Cancer PDX Tissues to Delineate Protein Interaction Network in Clinical Samples. *Journal of proteome research* **23**, 3269-3279 (2024).

8. Søndergaard CR, Olsson MH, Rostkowski M, Jensen JH. Improved treatment of ligands and coupling effects in empirical calculation and rationalization of p K a values. *Journal of chemical theory and computation* **7**, 2284-2295 (2011).

9. Kao A, et al. Development of a novel cross-linking strategy for fast and accurate identification of cross-linked peptides of protein complexes. *Molecular & Cellular Proteomics* **10**, (2011).

10. Yu C, Huang L. Cross-linking mass spectrometry (XL-MS): An emerging technology for interactomics and structural biology. *Analytical chemistry* **90**, 144 (2018).
