# Supplementary material for: Cysteine-enabled cleavability to advance cross-linking mass spectrometry for global analysis of endogenous protein-protein interactions
Source: Nat Commun. 2025 Dec 12;16:11093. doi: 10.1038/s41467-025-66023-0 (PMC12701074; doi:10.1038/s41467-025-66023-0)
Supplement: Supplementary file 2 — Description of Additional Supplementary Files [file 41467_2025_66023_MOESM2_ESM.pdf]

Description of Additional Supplementary Files

File Name: Supplementary Data 1

Description: Summary of the Reproducible SIA, SIAB and SBAP Cross-links of BSA Identified by LC MS^n

File Name: Supplementary Data 2

Description: Summary of the Reproducible SIA, SIAB and SBAP Cross-links of ALDOA Identified by LC MS^n

File Name: Supplementary Data 3

Description: Detailed Summary of the SIA, SIAB and SBAP Cross-linked Peptides Identified by LC MS^n from HEK 293 Cells

File Name: Supplementary Data 4

Description: Detailed Summary of the SIA, SIAB and SBAP Cross-linked Peptides Identified by LC MS^2 from HEK 293 Cells

File Name: Supplementary Data 5

Description: Summary of the Unique SIA, SIAB and SBAP Cross-links and Their Distance Mapping on Corresponding PDB Structures and/or AlphaFold Models

File Name: Supplementary Data 6

Description: Summary of CORUM Protein Complexes Present in the K-C XL-proteome and Their Subunit Recovery, and PDB Structure-resolved Protein Complexes Mapped by K-C Cross-links
