# Supplementary material for: Cysteine-enabled cleavability to advance cross-linking mass spectrometry for global analysis of endogenous protein-protein interactions
Source: Nat Commun. 2025 Dec 12;16:11093. doi: 10.1038/s41467-025-66023-0 (PMC12701074; doi:10.1038/s41467-025-66023-0)
Supplement: Supplementary file 9 — Reporting Summary [file 41467_2025_66023_MOESM9_ESM.pdf]

nature portfolio

Corresponding author(s): Lan Huang

Last updated by author(s): Oct 17, 2025

Reporting Summary

Nature Portfolio wishes to improve the reproducibility of the work that we publish. This form provides structure for consistency and transparency in reporting. For further information on Nature Portfolio policies, see our Editorial Policies and the Editorial Policy Checklist.

Statistics

For all statistical analyses, confirm that the following items are present in the figure legend, table legend, main text, or Methods section.

n/a	Confirmed	
	x	The exact sample size (*n*) for each experimental group/condition, given as a discrete number and unit of measurement
	x	A statement on whether measurements were taken from distinct samples or whether the same sample was measured repeatedly
x		The statistical test(s) used AND whether they are one- or two-sided<br>*Only common tests should be described solely by name; describe more complex techniques in the Methods section.*
x		A description of all covariates tested
x		A description of any assumptions or corrections, such as tests of normality and adjustment for multiple comparisons
	x	A full description of the statistical parameters including central tendency (e.g. means) or other basic estimates (e.g. regression coefficient) AND variation (e.g. standard deviation) or associated estimates of uncertainty (e.g. confidence intervals)
x		For null hypothesis testing, the test statistic (e.g. *F*, *t*, *r*) with confidence intervals, effect sizes, degrees of freedom and *P* value noted<br>*Give P values as exact values whenever suitable.*
x		For Bayesian analysis, information on the choice of priors and Markov chain Monte Carlo settings
x		For hierarchical and complex designs, identification of the appropriate level for tests and full reporting of outcomes
x		Estimates of effect sizes (e.g. Cohen's *d*, Pearson's *r*), indicating how they were calculated

Our web collection on statistics for biologists contains articles on many of the points above.

Software and code

Policy information about availability of computer code

Data collection	Orbitrap Fusion Lumos Tune Application (v3.5.3890), Xcalibur (v4.5.445.18)
Data analysis	Peak lists were extracted using PAVA, an in-house software developed by the UCSF Mass Spectrometry Facility.<br>For MSn analysis, extracted MS3 Peak lists were searched against a SwissProt human database (SwissProt.2021.10.12; 20,387 entries) using Protein Prospector (v6.3.5; http://prospector.ucsf.edu/prospector/mshome.htm).<br>The in-house software XL-Tools was used to automatically identify, summarize and validate cross-linked peptides based on Protein Prospector database search results and MSn data (Wang, et al. Mol Cell Proteomics 2017, 16 (5), 840-854).<br>For MS2 analysis, extracted MS2 peak lists were searched a database consisting of proteins identified from DSSO, DBrASO, and SIA MS3 analysis using a developmental version of Protein Prospector (v6.4.29).<br>Gephi (v0.10.1) was used to visualize XL-PPI network. ChimeraX (v1.2.5) used to map specific cross-links to 3-D structures.<br>The AlphaFold structure models were generated by applying the AlphaFold-Multimer (AFMv2.3, model 1) with default parameters and without templates. CombFold algorithm was used for integrative assembly of large complexes.

For manuscripts utilizing custom algorithms or software that are central to the research but not yet described in published literature, software must be made available to editors and reviewers. We strongly encourage code deposition in a community repository (e.g. GitHub). See the Nature Portfolio guidelines for submitting code & software for further information.

Data

Policy information about availability of data

All manuscripts must include a data availability statement. This statement should provide the following information, where applicable:

- Accession codes, unique identifiers, or web links for publicly available datasets
- A description of any restrictions on data availability
- For clinical datasets or third party data, please ensure that the statement adheres to our policy

All the in vivo SIA, SIAB, SBAP cross-linked peptide identifications are reported in Supplemental Data.
The mass spectrometry proteomics data have been deposited to the ProteomeXchange Consortium via the PRIDE partner repository with the dataset identifier PXD055169 (Reviewer account details: Username: reviewer_pxd055169@ebi.ac.uk Password: rO1OeQJVghtx).
For comparing in vivo K-C XL results to published large-scale XL-MS studies, we used:
- Jiao, F. , et al. Anal Chem 2022, 94 (10), 4236-4242.
- Wheat, A. , et al. Proc Natl Acad Sci U S A 2021, 118 (32).
- Jiao, F., et al. Anal Chem 2023, 95 (4), 2532-2539.
- Yugandhar, K., et al. Mol Cell Proteomics 2020, 19 (3), 554-568.
- Liu, F., et al. Mol Cell Proteomics 2018, 17 (2), 216-232.
- Bartolec, T. K, et al. Proc Natl Acad Sci U S A 2023, 120 (17), e2219418120.
- Jiao, F., et al. Journal of Proteome Research 2024, 23 (8),3269-3279.
For validating specific K-C cross-linking distances using ChimeraX, the following publicly available PDB structures were used:
- Bovine Serum Albumin (4F5S)
- Human EIF4E-EIF4G complex (5T46)
- Human aldolase A wild type (6XMH)
- FAF1 UBL1_L-Hsp70 NBD with ADP and phosphate (7FGM)
- SFPQ-NONO complex (7PU5)
- USP14-bound human 26S proteasome in state EA1_UBL (7W37)
- Prefoldin-tubulin-TRiC complex (7WU7)
- Human TRiC complex in closed state with nanobody bound (7NVL)
- Rabbit aldolase (8EW2)

Research involving human participants, their data, or biological material

Policy information about studies with human participants or human data. See also policy information about sex, gender (identity/presentation), and sexual orientation and race, ethnicity and racism.

Reporting on sex and gender	N/A
Reporting on race, ethnicity, or other socially relevant groupings	N/A
Population characteristics	N/A
Recruitment	N/A
Ethics oversight	N/A

Note that full information on the approval of the study protocol must also be provided in the manuscript.

Field-specific reporting

Please select the one below that is the best fit for your research. If you are not sure, read the appropriate sections before making your selection.

☒ Life sciences ☐ Behavioural & social sciences ☐ Ecological, evolutionary & environmental sciences

For a reference copy of the document with all sections, see nature.com/documents/nr-reporting-summary-flat.pdf

Life sciences study design

All studies must disclose on these points even when the disclosure is negative.

Sample size	There are no population studies so no sample size calculation was performed. Three bilogical replicates were chosen for our experiments to ensure reproducibility.
Data exclusions	No data excluded
Replication	We performed four replicates of SIA, six replicates of SIAB (three at room temperature and three at 37°C), and three replicates of SBAP analyzed by MS3. Additionally, we included two more SIA replicates (R3 and R4), three room-temperature SIAB replicates, and three SBAP replicates analyzed by MS2. All attempts at replication were successful.

Randomization	N/A
Blinding	N/A

Reporting for specific materials, systems and methods

We require information from authors about some types of materials, experimental systems and methods used in many studies. Here, indicate whether each material, system or method listed is relevant to your study. If you are not sure if a list item applies to your research, read the appropriate section before selecting a response.

Materials & experimental systems

n/a	Involved in the study
☐	☒ Antibodies
☐	☒ Eukaryotic cell lines
☒	☐ Palaeontology and archaeology
☒	☐ Animals and other organisms
☒	☐ Clinical data
☒	☐ Dual use research of concern
☒	☐ Plants

Methods

n/a	Involved in the study
☒	☐ ChIP-seq
☒	☐ Flow cytometry
☒	☐ MRI-based neuroimaging

Antibodies

Antibodies used	Streptavidin-HRP (Thermofisher Cat # N100)
Validation	The antibody used was a commercial antibody that has been validated by the company. The western results correlated well with the expected size of the protein. The dilution used was 1:10,000.

Eukaryotic cell lines

Policy information about cell lines and Sex and Gender in Research

Cell line source(s)	HEK293 cell lines stably expressing HTBH-tagged CSN6
Authentication	The cell lines have been autheticated using affinity purification and western blot analysis.
Mycoplasma contamination	Cells used in this study tested negative for mycoplasma.
Commonly misidentified lines (See ICLAC register)	N/A

Plants

Seed stocks	N/A
Novel plant genotypes	N/A
Authentication	N/A
